# Tricuspid Regurgitation in Patients with Tetralogy of Fallot

**DOI:** 10.3390/jcm12072470

**Published:** 2023-03-24

**Authors:** Christopher DeZorzi, Anais Marenco, Anne Marie Valente

**Affiliations:** 1 Department of Cardiology, Boston Children’s Hospital, Harvard Medical School, Boston, MA 02115, USA; 2 Department of Medicine, Division of Cardiology, Brigham and Women’s Hospital, Harvard Medical School, Boston, MA 02115, USA

**Keywords:** tetralogy of Fallot, tricuspid regurgitation, congenital heart disease

## Abstract

Tricuspid regurgitation in patients with repaired tetralogy of Fallot is an important finding with a wide spectrum of primary and secondary etiologies. Moderate or severe tricuspid regurgitation is associated with a greater incidence of atrial tachyarrhythmias in these patients. It remains uncertain which patients with repaired tetralogy of Fallot may benefit from a tricuspid valve intervention at the time of pulmonary valve replacement.

## 1. Introduction

In patients with repaired tetralogy of Fallot (rTOF), there has been significant focus on residual pulmonary regurgitation (PR) leading to right ventricular (RV) dilation and dysfunction commonly seen after an initial tetralogy of Fallot (TOF) repair. Over the past two decades, research efforts have largely been directed at determining the risk factors for adverse outcomes associated with PR and developing subsequent guidelines addressing the timing of intervention on the pulmonary valve in patients with rTOF. There has been less focus on the pathophysiology and contribution to adverse clinical outcomes of tricuspid regurgitation (TR). In this article, we review the mechanisms of TR in patients with rTOF as well as current data regarding intervention on tricuspid valve at the time of surgical pulmonary valve replacement (PVR). We also explore transcatheter interventions of tricuspid valve and describe the associations between tricuspid regurgitation and atrial arrhythmias in patients with rTOF.

## 2. Mechanisms of Tricuspid Regurgitation

**Figure 1:**
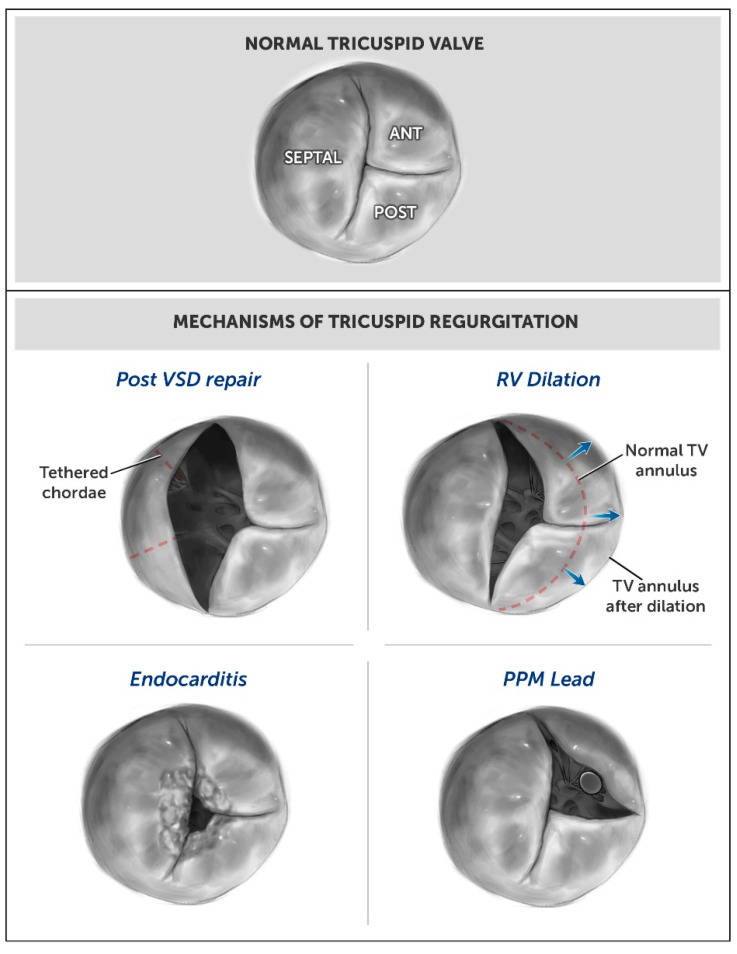
Mechanisms of Tricuspid Regurgitation in Patients with Repaired Tetralogy of Fallot. (ANT–anterior, POST–posterior, VSD–ventricular septal defect, RV–right ventricle, TV–tricuspid valve, PPM–permanent pacemaker).

There are several potential underlying mechanisms of TR in patients with rTOF. We have categorized these into two distinct groups, primary and secondary TR, in order to be consistent with standard valvular nomenclature (see Figure 1). We will discuss both groups separately below, with further division of the primary TR category into acquired and congenital etiologies.

For the purpose of this article, we describe TR as primary when the etiology of the regurgitation is due to a structural defect or damage to the valve tissue or apparatus, leading to a backward flow of blood from the RV to the right atrium during ventricular systole. In patients with rTOF, this will most commonly be an acquired etiology secondary to the disruption of the integrity of the septal leaflet of the tricuspid valve during the placement of the ventricular septal defect (VSD) patch in an initial repair. This annular dehiscence results in a TR jet originating at the junction between the VSD patch and the septal attachment of the tricuspid valve, extending along the atrial septum. The operative approach to VSD repair does not seem to be associated with the development of significant TR. In one study with 133 rTOF patients, moderate or severe TR was found in 13–25% of the patients regardless if the VSD was closed through the right atrium and tricuspid valve (n = 66), through the pulmonary artery (n = 15), or through the RV (n = 52) [1].

Other causes of acquired primary TR include endocarditis, rheumatic heart disease, carcinoid syndrome, and trauma. Trauma to the valve leaflet chordae can be from an external chest wall trauma or intracardiac procedure, such as pacemaker or defibrillator lead implantation, myocardial biopsy, or prior surgery. Pacemaker leads, which disrupt the tricuspid valve leaflets coaptation, commonly cause primary TR. Primary TR can also be congenital due to intrinsic abnormalities of the valve from birth. Although possible, intrinsic congenital abnormalities of the valve in combination with TOF are rare.

We define secondary TR as when the tricuspid valve leaflets and apparatus are structurally normal, but extrinsic factors cause changes in the competence of the tricuspid valve and its ability to keep blood from returning to the right atrium during ventricular systole. Many patients have residual severe PR when a transannular patch repair is required early in life to relieve congenital right ventricular outflow tract (RVOT) stenosis. In patients with rTOF, severe PR results in progressive RV dilation, which leads to secondary TR due to annular dilation. The dilated ventricle alters the chamber geometry from the basal–lateral displacement of the free wall papillary muscles, which leads to a distorted subvalvular apparatus. Displacement of the papillary muscles can also be a cause of secondary TR independently or in combination with RV dilation. Although dilation of the RV, TV annulus, and RA are commonly referred to as an etiology of TR, Offen et al. found poor correlation between RV volume, right atrial volumes, and tricuspid annular dilatation with the presence of significant TR [2].

Aside from the TV annular dehiscence described as primary TR, scarring and tethering of the septal leaflet to the VSD patch is common at a later stage after repair and considered a secondary cause of TR. Patients with left-sided heart disease, including left ventricular systolic and diastolic dysfunction, can also have secondary TR. Pulmonary hypertension from any cause may also contribute to secondary TR [3,4].

## 3. Surgical Interventions on Tricuspid Valve in Repaired TOF

Over the past two decades, there have been a number of studies reporting tricuspid valve interventions at the time of PVR in patients with rTOF; however, most studies are limited by a small cohort size with a relatively short duration of follow-up (Table 1). While the findings are not entirely consistent, many have shown improvement in TR regardless of whether or not there is intervention on the tricuspid valve at the time of PVR. In 2010, a study by Kogon et al. of 35 subjects with RVOT lesions (26 rTOF and 9 congenital pulmonary stenosis) and at least moderate TR demonstrated no difference in the degree of TR postoperatively between those patients who underwent PVR alone (n = 19) and those who underwent concomitant tricuspid annuloplasty (n = 16) [5]. However, after a mean of seven years post-procedure, the degree of tricuspid regurgitation was significantly higher in the concomitant annuloplasty group [6]. In a 2015 study that included 36 patients with rTOF who had at least moderate TR and underwent either PVR with concomitant tricuspid intervention (n = 18) or PVR alone, Cramer et al. suggested there was significant improvement in the degree of TR and RV size at six months following PVR, regardless of whether tricuspid valve repair was performed [7]. In a report on patients with rTOF who had tricuspid valve repair during PVR for the indication based solely on annular dilation (greater than 40 mm), patients who underwent tricuspid repair (n = 10) had a longer length of stay in intensive care compared to those without tricuspid valve intervention (n = 18). Importantly, postoperative echocardiography showed recovery of the RV function, regardless of whether or not concomitant tricuspid valve repair was performed [8]. 

The preoperative degree of TR may have a significant impact on whether patients will have better outcomes with PVR alone rather than in combination with tricuspid valve repair. Interestingly, in a study by Roubertie et al., patients with rTOF with moderate TR prior to surgery had similar results to the studies above, but patients who had severe TR prior to surgery had worse outcomes. In the subgroup of patients who had severe TR prior to surgery, but did not undergo tricuspid valve repair, significant (moderate or greater) residual TR was reported in seven of eight patients. Among the seven patients with residual TR, two required a tricuspid valve replacement. Additionally, there was improvement in the New York Heart Association (NYHA) Functional Class in those who had concomitant tricuspid valve repair [9]. More recently in 2019, Deshaies et al. demonstrated that tricuspid valve intervention at the time of PVR was associated with an additional 2.3-fold reduction in TR grade without an increase in early adverse events or hospitalization time, at the three-month follow-up [10].

Tricuspid valve intervention may not be protective against future TR in patients with pulmonary valve failure, defined as ≥ moderate PR, or pulmonary stenosis with peak velocity of more than 3.5 m/s. Min et al. in 2022 described 33 rTOF patients with moderate or less TR who underwent tricuspid valve repair at the same time as PVR. In patients who had pulmonary valve failure, there was no protection against TR, even in those patients who had a concomitant tricuspid valve repair [11]. 

**Table 1:** Studies comparing pulmonary valve replacement alone vs. pulmonary valve with concomitant tricuspid valve intervention in rTOF.

Reference	Institution	Year	Study Design	Total Subject # (TVI Subject #)	Follow-Up	Summary
Kogon et al. [5,6]	Emory University, Atlanta, GA, USA	2010 & 2015	Retrospective	35 (16)	7 (± 2.8) years	TR was significantly higher in the PVR + TVI (annuloplasty) group at long-term follow-up.
Cramer et al. [7]	MCW, Milwaukee, WI, USA	2015	Retrospective	36 (18)	6 months	Significant improvement in TR and right ventricular size after PVR without statistical differences between those patients undergoing PVR vs. PVR+TVI.
Jones et al. [12]	Multicenter (10 centers)	2016	Retrospective	300 (0)	5 years	After TPVR in patients with at least moderate TR at baseline, TR severity improved in 65% of patients.
Roubertie et al. [9]	Bordeaux University, Bordeaux, France	2017	Retrospective	104 (16)	5 years	PVR+TVI might improve both tricuspid valve function and functional status of patients in cases of severe preoperative TR.
Lueck et al. [8]	Muenster University, Muenster, Germany	2018	Retrospective	28 (10)	N/A	PVR+TVI, determined based on annular dilation >40 mm, led to increased ICU stay with more complications compared to PVR. Recovery of the RV function and diameters occurred independent of concomitant TVI.
Deshaies et al. [10]	Canadian Congenital Cardiac Collaborative	2020	Retrospective	542 (180)	3 months	PVR+TVI was associated with additive improvement in TR without an increase in early adverse events or hospitalization time.
Min et al. [11]	Seoul National University, Seoul, South Korea	2022	Retrospective	119 (33)	N/A	In patients with pulmonary valve failure, there was no protection against TR in patients with TVI.

PVR—pulmonary valve replacement; TVI—tricuspid valve intervention; MCW—Medical College of Wisconsin; TR—tricuspid regurgitation; ASD—atrial septal defect; USA—United States of America.

## 4. Transcatheter Interventions on Tricuspid Valve

Transcatheter interventions are increasingly used in the treatment of valvular heart disease, including in patients with congenital heart disease. When an intervention on the pulmonary valve is performed using a transcatheter approach, TR is improved in some but not all patients with rTOF. Jones et al. reported that transcatheter pulmonary valve replacement (TPVR) reduced TR in patients with RV volume and pressure overload. In patients with at least moderate TR at baseline, TR severity improved in 65% of the patients. Baseline moderate and severe TR was associated with shorter freedom from RVOT re-intervention after TPVR [12].

There are a growing number transcatheter options for the tricuspid valve. In patients with prior annuloplasty or bioprosthetic valve replacement, Melody and Sapien valves can be used for valve replacement. There are high rates of procedural success and immediate hemodynamic benefits [13]. Combined transcatheter tricuspid and pulmonary valve replacement has been reported in a case series of five patients [14]. Further evaluation of long-term outcomes is needed in this area.

In patients without congenital heart disease and with native valve leaflets and annulus, transcatheter edge-to-edge repair (TEER) has been successfully used in patients with heart failure, who have been optimized on medical therapy. The follow-up of the TRILUMINATE study (Trial to Evaluate Treatment With Abbott Transcatheter Clip Repair System in Patients With Moderate or Greater Tricuspid Regurgitation) showed that TR severity was significantly reduced (TR reduction of ≥1 grade in 87% of subjects, with 70% achieving moderate or less TR) at the one-year follow-up [15]. In the CLASP TR study (Edwards PASCAL Transcatheter Valve Repair System in Tricuspid Regurgitation), 85% of patients achieved a TR severity reduction of at least 1 grade, with 52% achieving moderate or less TR without increased mortality at the one-month follow-up [16]. Lastly, transcatheter tricuspid valve replacement has an exciting future with the ongoing development of the Cardiovalve (Boston Medical), Evoque (Edwards), and other valves [17].

## 5. Association of Arrhythmias and TR in Repaired TOF

Significant TR is associated with atrial tachyarrhythmias in patients with rTOF [18]. In a large registry to assess risk factors for arrhythmia and sudden cardiac death in patients with rTOF, PR was the main underlying hemodynamic lesion for patients with ventricular tachycardia and sudden death; however, TR was the underlying pathology for those with atrial flutter/fibrillation [19]. It is unknown what the long-term arrhythmic outcomes are in patients who receive concomitant tricuspid valve interventions during PVR versus those who do not. 

In 2010, the Alliance for Adult Research in Congenital Cardiology assessed 556 adult patients with rTOF, showing the prevalence of atrial tachyarrhythmias was 20.1%. In rTOF patients with atrial arrhythmias, 32.6% had at least moderate TR, whereas only 10.8% of patients without arrhythmia had at least moderate TR [20]. In a patient population of 303 patients with rTOF at the Mayo Clinic, which had a similar prevalence of atrial arrhythmia (24%), 47% of patients with atrial fibrillation had at least moderate TR, whereas only 11% of patients who had no arrhythmia had at least moderate TR. Notably, atrial fibrillation was also associated with reduced 20-year transplant-free survival, even after the adjustment for differences in age [21].

## 6. Summary

- There has been less focus on the pathophysiology and contribution to adverse clinical outcomes of TR compared to PR in patients with rTOF.
- Primary TR in patients with rTOF, defined as a structural defect or damage to the valve tissue or apparatus, usually results from the disruption of the integrity of the septal leaflet of the tricuspid valve during the placement of the VSD patch in an initial repair, endocarditis, or after pacemaker lead implantation.
- Secondary TR in patients with rTOF results from progressive RV dilation and annular dilation (commonly seen with longstanding PR).
- Over the past two decades, although there have been a number of studies reporting tricuspid valve interventions at the time of PVR in patients with rTOF, there is lack of evidence to recommend concomitant tricuspid valve intervention for the majority of patients.
- After TPVR, improvement in TR grade is seen in some but not all patients with rTOF.
- There are a growing number transcatheter options for tricuspid valve.
- Atrial tachyarrhythmias are increasingly common in patients with rTOF as they age, and those with ≥ moderate TR have a greater likelihood of atrial fibrillation.

## Data Availability

Not applicable.
